# Epidemiological characteristics and spatio-temporal analysis of brucellosis in Shandong province, 2015–2021

**DOI:** 10.1186/s12879-023-08503-6

**Published:** 2023-10-09

**Authors:** Xiaolin Yu, Ming Fang, Yan Li, Jianmei Yu, Lixiao Cheng, Shujun Ding, Zengqiang Kou

**Affiliations:** 1 Institute for Communicable Disease Control and Prevention, Shandong provincial Center for Disease Control and Prevention, Jinan, Shandong China; 2 Department of public health and health management, Shandong First Medical University, Jinan, Shandong China

**Keywords:** Brucellosis, Epidemiological characteristics, Spatial autocorrelation, Spatio-temporal cluster analysis

## Abstract

**Background:**

Brucellosis is one of the major public health problems in China, it not only causes huge economic losses to the society, but also threatens the human’s physical and mental health. The reported cases of brucellosis in Shandong province were at a high level, therefore, it is necessary for us to understand the epidemic characteristics and distribution trend of Brucellosis in Shandong province. This study aims to describe the epidemiological characteristics and spatial clustering characteristics of brucellosis in Shandong Province, provide a reference for the scientific prevention and control.

**Methods:**

Human brucellosis data in Shandong province from 2015 to 2021 were obtained from the China Information System for Disease Control and Prevention, the data were analyzed by descriptive epidemiological methods, spatial autocorrelation analysis and spatial-temporal cluster analysis methods use ArcGIS and SaTScan software, the results were presented in ArcMap.

**Results:**

A total of 22,251 human cases of brucellosis were reported, the annual incidence ranged between 2.41/100,000 and 4.07/100,000 from 2015 to 2021 in Shandong province, incidence has been decreasing year by year, while there was a significant increase in 2021. The distribution of brucellosis was of a seasonal trend, mainly concentrating during March to August. The age of the cases was mainly concentrated in the 30–74 age ranges, the average annual incidence rate was significantly higher in males than in females. The spatial analysis showed that the epidemics were mainly concentrated in the north and southwest. For the spatial autocorrelation analysis, a high global autocorrelation was observed at the county level, and the high–high clusters mainly distributed in the north and southwest region. For the spatio-temporal scanning, the most likely cluster areas mainly distributed in the north area, and then gradually moved southward, and the radius of clustered narrowed.

**Conclusions:**

Human brucellosis remains a common challenge, particularly in northern region in spring and summer. More disease prevention and control measures should be taken in high-risk populations, and such higher-risk susceptible areas to reduce the incidence of brucellosis and ensure the health of the people.

## Background

Brucellosis is the most important zoonotic disease caused by Brucella species comprising Gram negative, facultative, intracellular pathogens, [1, 2]. Domestic animals such as cattle, goats, sheep, pigs, camel, buffalo and dogs serve as a reservoir hosts [3]. The disease is transmitted to humans by direct/indirect contact with infected animals or through the consumption of raw meat and dairy products. The main transmission routes are digestive tract, skin, and mucosal and respiratory tract contact with blood body fluids and aerosols [4]. The impact of brucellosis on human health is a major issue, it cause fever, nausea, muscular pain, abdominal pain, sweating, weakness, decreased appetite, weight loss, and liver inflammation [1]. Brucellosis not only causes huge economic losses to the society, but also threatens the human’s physical and mental health [5]. Human brucellosis is one of the major public health problems in China.

Brucellosis is prevalent worldwide, Cases of brucellosis have been reported in 170 countries and regions. There are about 50,000 to 6 million people with more than 500,000 human cases reported annually worldwide, the Middle East, Asia, the Mediterranean Basin, Africa, Central America and the Caribbean being the main endemic regions [6, 7]. The evolution of the “global village” through international tourism. new endemic foci have emerged [8], So far, China has 30 provinces (municipalities, autonomous regions) have different degrees of brucellosis epidemic, there are now 300,000 to 500,000 patients [9]. From the perspective of geographical distribution, the affected regions in China gradually expanded from the northern traditional pasturing regions to the agricultural areas and finally to the southern coastal and southwestern areas [10]. The reported cases of brucellosis in Shandong province are at a high level, with the number of reported cases averaging more than 2700 cases per year in the past three years. The situation of brucellosis prevention and control is grim. This study aims to describe the epidemiological characteristics and spatial clustering characteristics of brucellosis in Shandong Province, provide a reference for the scientific prevention and control of brucellosis.

## Methods

### Research data

The data of human brucellosis cases in Shandong province between 2015 and 2021were obtained from the China Information System for Disease Control and Prevention. The main contents of the database of human brucellosis include the number of cases, incidence rate, number of deaths and mortality rate in multiple dimensions by the region, age group, gender and occupation. The diagnostic criteria were based on the national health industry standard. The demographic information were obtained from the Shandong Statistical Bureau. Geographic space information was acquired from the National Geographic Information Public Service Platform.

### Statistical analysis

The SPSS software (version 23.0, IBM company, NewYork, USA) was used for the descriptive epidemiological analysis. *χ*^*2*^ test was used for comparison of count data, and the test level was α = 0.05. Spatial mapping and spatial autocorrelation analysis were based on Arcgis software(Version, 10.6, Environmental Systems Research Institute, Redlands, USA). SaTScan software(version 9.5, Boston, MA, USA) was used for spatial-temporal statistical.

### Spatial autocorrelation analysis

As a spatial statistical method, global spatial autocorrelation and local spatial autocorrelation are used to describe the relationship between study areas and measure the degree of clustered or dispersion [11–13]. The Moran’s I index is used to measure overall spatial autocorrelation and spatial distribution of the study areas while the local one can be further used to reflects the local spatial autocorrelation and the specific clustering areas [14]. The value of Moran’s I ranges between [− 1, 1], I > 0 indicates a positive spatial correlation, while I < 0 indicates a negative spatial correlation, if the I value is close to 0, indicates that the cases are randomly distributed in space, no spatial correlation exists [15, 16]. The larger the absolute value of I, the stronger the correlation. When Z > 1.96, P < 0.05 was considered to be statistically significant and a spatial autocorrelation existed [17, 18]. LISA is used to reflect a geographical phenomenon on a regional unit or the degree of correlation between an attribute value and the same geographical phenomenon or attribute value on the adjacent unit [19]. The spatial correlation patterns obtained from the local Moran’s I index can be shown by LISA, which are classified into four types ,low-high cluster (L-H, which indicated that the low cluster areas were surrounded by high cluster areas), high–low cluster (H-L, which indicated that the high cluster areas were surrounded by other low cluster areas), low-low cluster (L-L, which indicated the cold spot), and high-high cluster (H-H, which indicated the hot spot) [20, 21]. The spatial autocorrelation analysis was conducted by ArcGIS software.

### Spatio-temporal cluster analysis

We used the spatio-temporal scan statistics to detect the center and radius of the aggregation area [22, 23], and verify whether the time and geographic clustering of human brucellosis was caused by random variation or not [24]. The basic principles of spatio-temporal scan is based on a discrete Poisson model [25]. The theoretical incidence number of each scanning window is calculated and compared with the actual incidence number to construct the log likelihood ratio (LLR) [26], By calculating the LLR of the spatial unit attributes within and outside the dynamic window area under different centers and radiuses, SaTScan makes the statistical inference and explores the maximum possible clustering area using Monte Carlo for the statistical significance evaluation [27].For each possible spatial-temporal clustering area, when P < 0.05, the larger the log-likelihood ratio (LLR) value, the more the likelihood that the area covered by the scanning dynamic window represents the clustering area [28, 29]. Finally, the window with the largest LLR value is selected as the maximum possible clustering area, which represents this high-risk region [30], while other statistically significant windows were the secondary possible clustering area [31]. In spatio-temporal scan analysis, the spatial unit was set as county, the temporal unit was set as year. Circular moving windows were set to scan the study area. The default size settings for the window and time are usually set to 50% [32], The number of Monte Carlo randomization was set to 999.SatScan software was used for spatial-temporal statistical, and Arc GIS software was used for visual presentation of the scanning results.

## Results

### Demographic characteristics

From 2015 to 2021, the average annual incidence rate was 4.42/100,000 in males and 1.84/100,000 in females, which were significantly higher in males than in females (χ2 = 3142.31, P<0.01). The male-to-female ratio ranging from2.43:1 to 2.65:1. The incidence is generally higher in the age range of 30–74 years ranging from1.57/100,000 to 9.88/100,000, we found that the occupation distribution is mainly farmers, from 2015 to 2021, a total of 18,975 cases were reported, accounting for 85.28% of the total cases. (Table 1).

**Table 1:** Demographic characteristics of Brucellosis in Shandong province, 2015–2021

	2015	2016	2017	2018	2019	2020	2021
Annual incidence rate	3.86	4.07	3.25	2.83	2.52	2.41	3.32
Age (/100,000)							
0-	2.97	2.57	2.40	2.06	1.65	1.61	1.97
5-	2.96	1.53	1.61	2.37	2.00	1.05	1.15
10-	0.55	0.84	0.43	0.49	0.37	0.55	0.21
15-	0.89	1.54	0.93	0.98	0.41	0.75	0.42
20-	0.59	0.84	0.64	0.52	0.95	0.83	1.21
25-	2.67	3.50	2.64	1.96	1.15	0.83	1.05
30-	3.44	4.24	3.54	3.49	2.05	1.57	2.33
35-	3.62	3.94	3.11	2.60	2.57	2.34	3.17
40-	4.37	4.58	3.28	2.71	2.51	2.42	3.53
45-	4.90	5.69	4.64	3.81	3.81	2.80	4.02
50-	8.62	9.56	8.28	7.50	4.35	4.15	5.77
55-	6.89	6.13	4.48	4.45	5.30	4.93	7.57
60-	9.65	9.25	7.67	6.32	5.27	4.78	6.74
65-	9.88	8.40	6.40	5.47	4.60	5.43	7.53
70-	4.58	4.54	4.31	4.24	3.38	3.79	5.23
75-	1.65	1.92	1.70	1.68	1.80	2.44	2.62
80-	0.74	0.78	0.97	0.68	0.84	0.79	0.86
≥ 85	0.70	0.87	0.46	0.50	0.19	0.67	0.64
Gender (/100,000)							
Male	5.44	5.64	4.53	3.89	3.57	3.42	4.70
Female	2.23	2.43	1.90	1.73	1.42	1.35	1.91
Male-to-female ratio	2.5	2.43	2.5	2.36	2.63	2.65	2.53
Population classification (%)							
Children	1.32	1.02	1.08	1.34	1.34	1.07	0.95
Student	1.64	2.00	1.42	1.87	1.54	2.47	1.19
Medical workers	0.21	0.15	0.06	0.11	0.19	0.08	0.24
Workers	0.00	0.00	2.76	2.36	2.01	1.56	3.50
Farmer	85.14	85.73	87.64	84.84	86.74	84.75	84.01
Shepherd	0.77	0.32	0.22	0.28	0.47	0.45	0.77
Office clerk	2.20	2.47	1.89	1.52	1.77	1.32	1.54
Housework and unemployment	3.13	2.54	2.63	2.29	3.12	4.86	5.96
Others	5.59	5.76	2.29	5.39	2.82	3.44	1.84

### Temporal characteristics

**Fig. 1:**
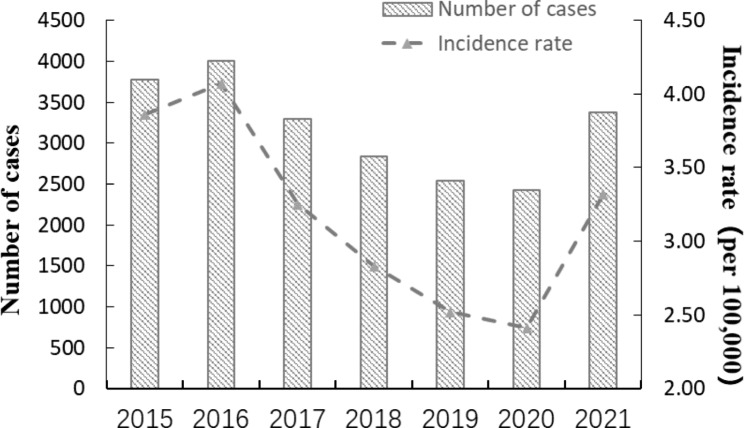
Reported cases and incidence rate of Brucellosis in Shandong province, 2015–2021

**Fig. 2:**
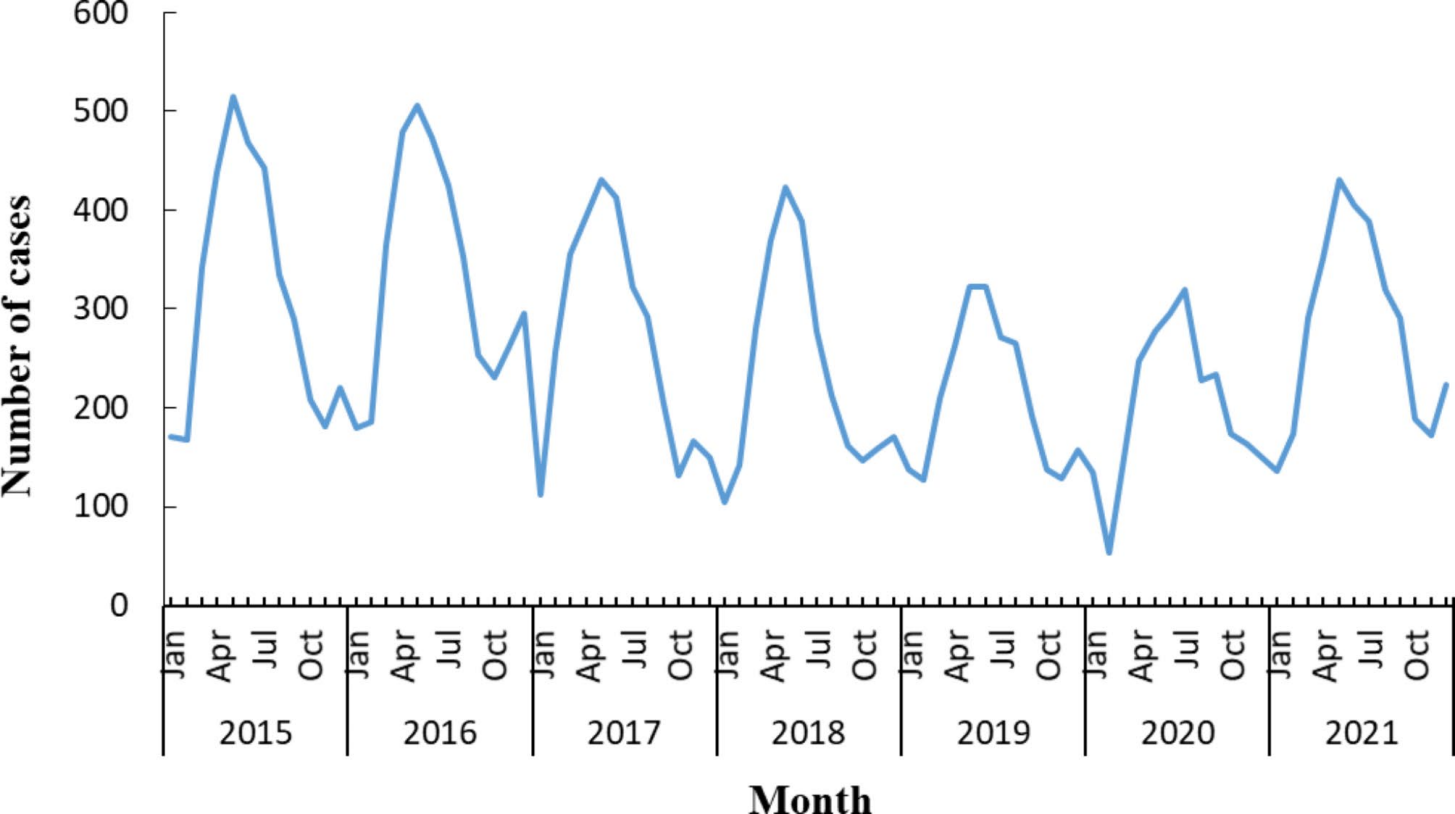
Monthly distribution of Brucellosis cases in Shandong province, 2015–2021

A total of 22,251 cases of brucellosis were reported in Shandong province from 2015 to 2021, and the annual reported incidence ranged between 2.41/100,000 and 4.07/100,000. Since 2015, the incidence of brucellosis in Shandong province has firstly increased and then decreased, and reached the peak in 2016, with reported incidence 4.07/100,000 From 2017 to 2020, brucellosis has been decreasing year by year, while there was a significant increase in 2021(Fig. 1). Brucellosis cases were reported every month throughout the year, with clear seasonal distribution characteristics. The peak of brucellosis incidence was concentrated from March to August, accounting for 66.08% of the total cases (Fig. 2).

### Spatial characteristics

**Fig. 3:**
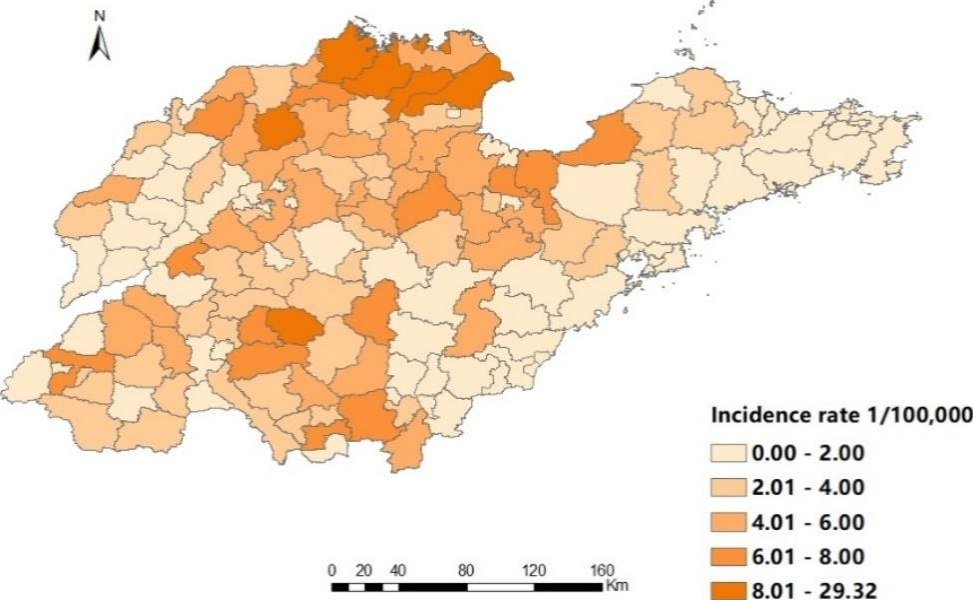
Regional distribution of annual incidence of Brucellosis in Shandong Province, 2015–2021

**Fig. 4:**
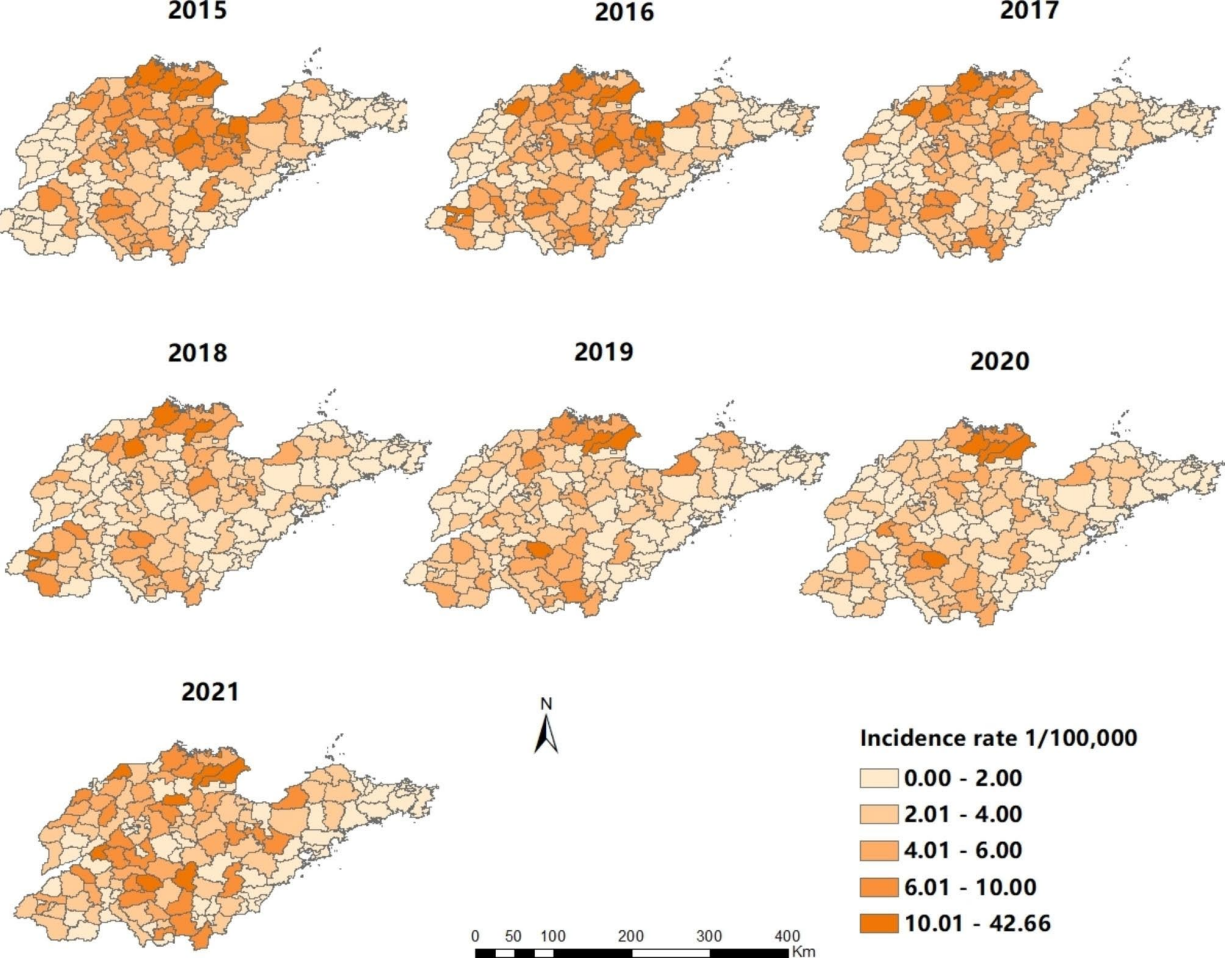
The yearly incidence of Brucellosis at the county level in Shandong province, 2015–2021

Brucellosis cases were reported in 16 cities of Shandong province from 2015 to 2021, and the number of counties (districts) affected by the epidemic was 123, 122, 122, 140, 137, 140 and 140 respectively. The top five counties (districts) with average annual incidence were Lijin County (29.32/100 000), Kenli County (12.39/100 000), Sishui County (10.92/100 000), Wudi County (9.85/100 000) and Zhanhua County (9.63/100 000), mainly located in the north and southwest of Shandong Province (Fig. 3). In space, there is a gradual narrowing of the central epidemic area. In general, the incidence rates in most areas have been decreasing year by year, especially in the central area (Fig. 4).

### Spatial autocorrelation analysis

**Fig. 5:**
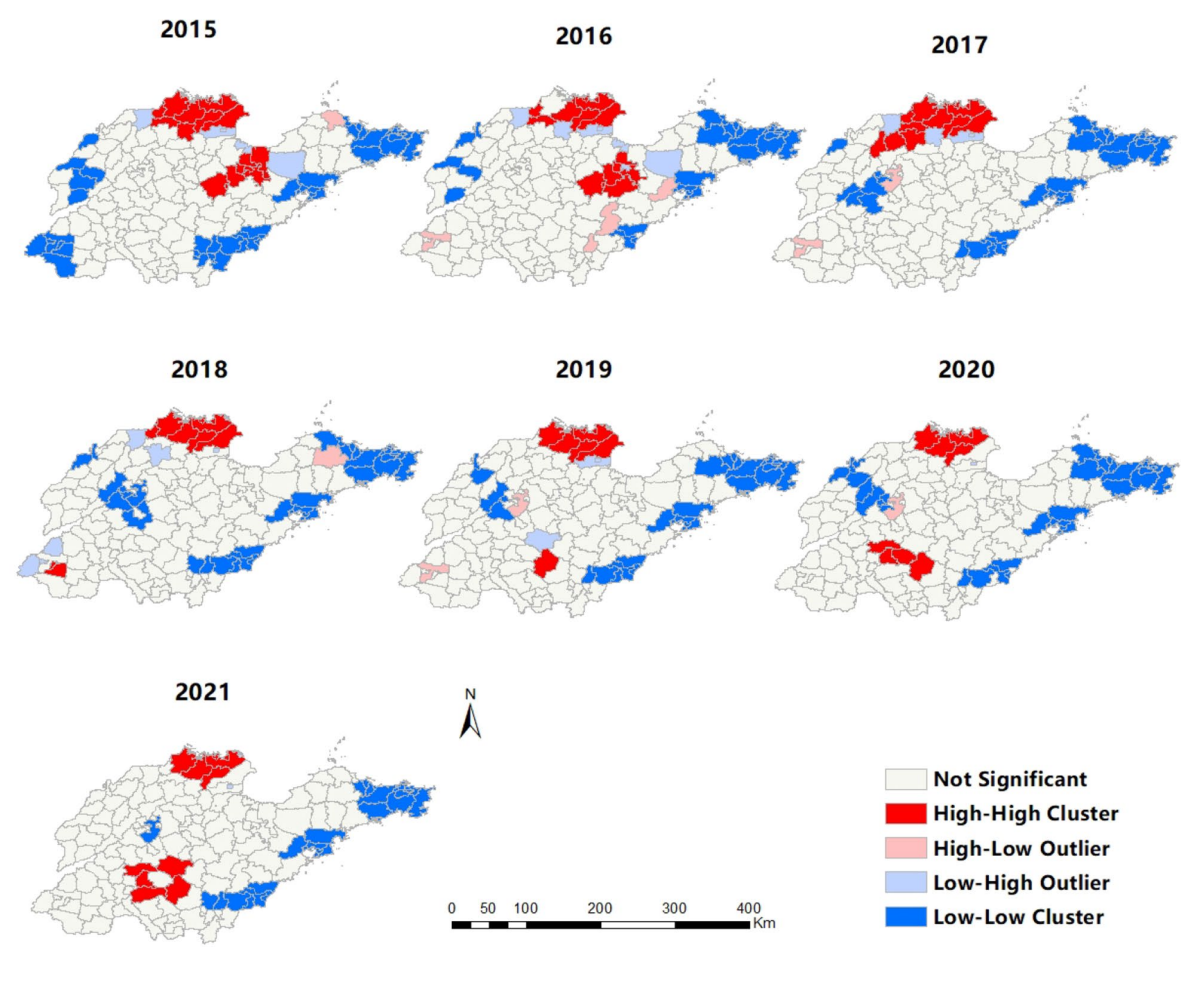
Results of local spatial autocorrelation analysis of Brucellosis at the county level in Shandong province, 2015–2021

The global spatial autocorrelation analysis found that the annual global Moran’s I values ranged from 0.205 to 1.547, while the Z values were greater than 1.96 (all p < 0.001), which indicates that the incidence of brucellosis between 2015 and 2021 had a positive spatial autocorrelation and obvious spatial clustered distribution at the county level, the highest clustering degree was in 2020 (Moran’s I = 1.547) and the lowest in 2016 (Moran’s I = 0. 205) (Table 2). Local autocorrelation analysis results were shown in Fig. 5. The LISA clustered distribution map showed that there were15, 12,10, 8, 6,8 and9 counties (districts) in the high-high clustered area from 2015 to 2021, and the “high-high” clustered area was mainly concentrated in the northern part of the province. Located in Distributed in Hekou District, Lijin County, Kenli District, Wudi County, Yangxin County, Huimin County, Zhanhua County, Bincheng District, Qingyun County, Shanghe County, Pingyi County, Shouguang city, Sishui County, Qufu City, Xintai City, Ningyang County and other regions. Hekou District, Lijin County and Zhanhua County are the “high-high” gathering areas over the years. Over time, the number of counties (districts) covered by high-high clustered areas showed a downward trend. In recent years, Pingyi County of Linyi City, Ningyang County of Taian city and Qufu City of Jining became the new “high-high” clustered areas. The “low-low” clustered area area became smaller year by year and its distribution changed to concentrated in the eastern region.

**Table 2:** Results of the spatial autocorrelation test on Brucellosis cases in Shandong province, 2015–2021

Year	Moran’s I	Z score	P value	clustered
2015	0.270	4.142	0.001	YES
2016	0.205	3.655	0.001	YES
2017	0.308	3.998	0.001	YES
2018	0.742	8.161	0.001	YES
2019	0.829	8.973	0.001	YES
2020	1.547	16.441	0.001	YES
2021	0.288	3.181	0.001	YES

### Spatio-temporal cluster analysis

**Fig. 6:**
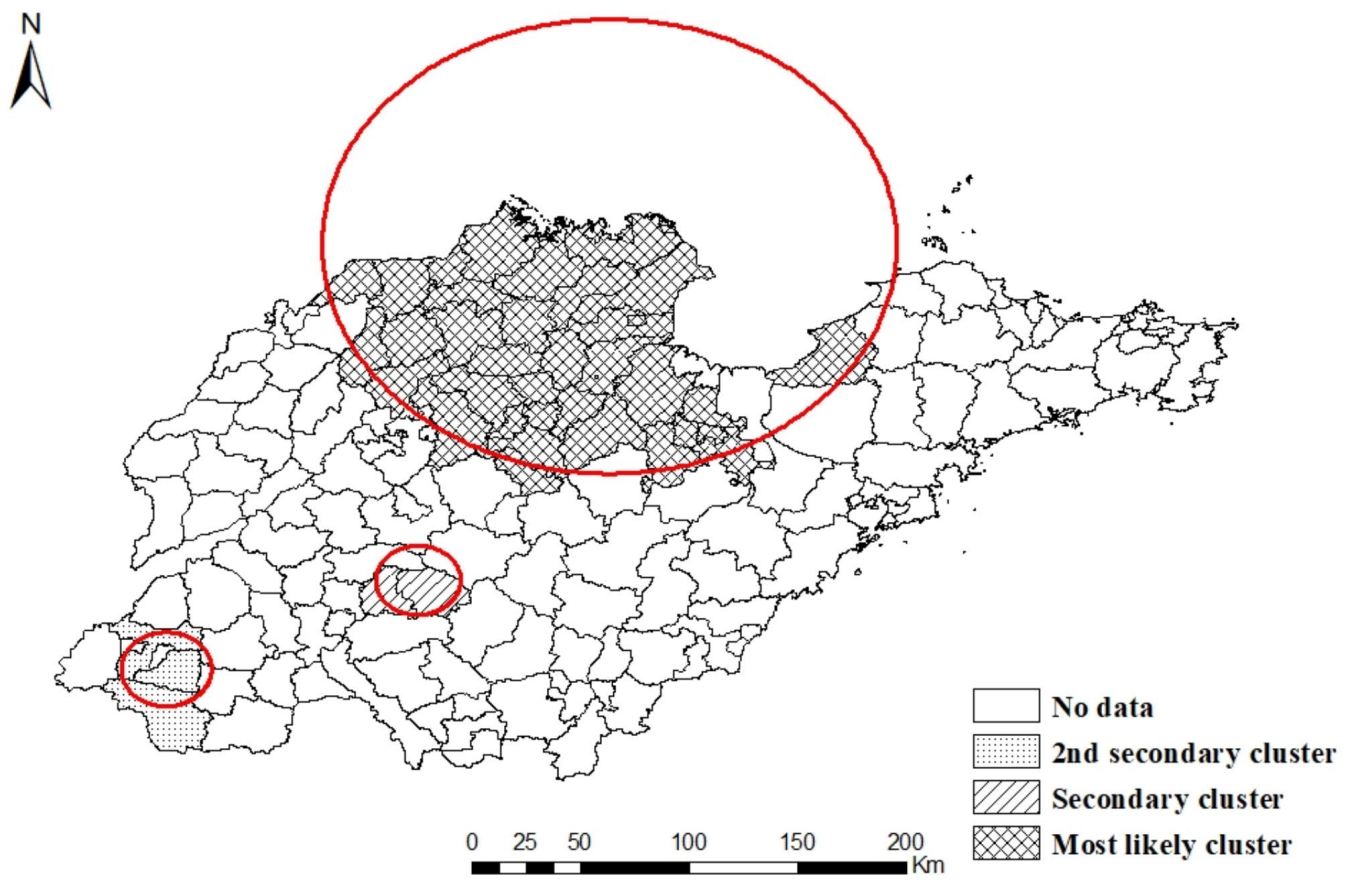
Spatio-temporal clustering of brucellosis cases in Shandong province from 2015to 2021. (The red circles represent the clustering areas, such that the largest represents the most likely cluster area.)

The results of the spatio-temporal cluster analysis for the human brucellosis cases in Shandong province from 2015 to 2021 included one most likely cluster, one secondary cluster and one 2nd secondary cluster. The most likely cluster was located in the northern area, including 37 counties and districts, of which the cluster center was (37.88 N,118.52E) and the cluster radius was 167.91 km. and the time frame was from January 2015 to December 2017, with a relative risk (RR) value of 2.52 (P < 0.0001). The secondary clusters were relatively smaller, mainly distributed in the central area, including Qufu City, Sishui County, of which the cluster center was (35.66 N, 117.25E) and the cluster radius was25.56 km. and the cluster time frames mainly ranged from January 2019 to December 2021 ,with a relative risk (RR) value of 4.17 (P < 0.0001). The 2nd secondary clusters were mainly distributed in the southwest area of Shandong province, including Dingtao District, Mudan district, Cao County, and the cluster time frames mainly ranged from January 2016 to December 2018 with a relative risk (RR) value of 2.59 (P < 0.0001) (Table 3; Fig. 6).

**Table 3:** Spatio-temporal clustering of human Brucellosis in Shandong province, 2015–2021

Cluster	Cluster areas	Cluster center/radius(km)	Period	Number of cases	Expected cases	RR	LLR	P value
Most likely cluster	37	37.88 N,118.52E/167.91	2015/1/1 to 2017/12/31	5498	2514	2.52	1526.24	< 0.0001
Secondary cluster	2	35.66 N, 117.25E/ 25.56	2019/1/1 to 2021/12/31	412	100	4.17	273.19	< 0.0001
2nd secondary cluster	3	35.07 N, 115.57E/ 27.39	2016/1/1 to 2018/12/31	709	278	2.59	236	< 0.0001

## Discussion

Brucellosis is one of the most widespread zoonoses worldwide [7, 33], In China brucellosis has been increasingly causing huge economic loss, and it has been a public health problem in recent years [4]. Public health researchers are usually interested in using data visualization methods to describe the distribution of disease. It can guide public health officers and medical providers to fast response and make decision about containment, management, and prevention of diseases, to improve the surveillance and control strategies [34, 35]. In this study, the incidence of brucellosis at the county level in Shandong province from 2015 to 2021 was used to discuss the epidemiological characteristics and investigate its spatial and temporal distribution rules and possible clustered areas. Better display of incidence and spatial clustered through map visualization, this study provides the theoretical support for the prevention and control of human brucellosis.

From 2005 to 2014, the incidence of brucellosis in China was on the rise, with the reported incidence rising from 1.40/100,000 to 4.22/100,000, and then the incidence was on the decline from 2015 to 2017 [36]. The brucellosis epidemic in Shandong province is slightly different from that in the whole country, showing an upward trend and then a downward trend since 2015 to 2020, and an upward trend in 2021. It peaked in 2016 with incidence rate of 4.07 per 100,000. Shandong province issued the corresponding strengthening of prevention and control policies, to suppress brucellosis epidemic rising trend played a positive role. The increase in 2021 May be associated with abundant rain in 2021, which is more conducive to the propagation and spread of pathogens in the high-incidence season, the spread of infectious diseases with natural foci is affected by rainfall [37].

Our study found that the higher incidence was concentrated in the 30–74 age ranges, most of the people working with livestock are middle-aged, within this age range. In rural Chinese families, most middle-aged and elderly people aged between 40 and 65 are raising livestock [38], so the incidence of brucellosis among this age group is higher. Drinking unpasteurized goat milk or cow’s milk can also lead to brucellosis infection [39, 40], infection in other age groups may be associated with drinking raw milk or other means of exposure to infected livestock or their products. More males than females are affected, because of there are more men in jobs that have more contact with livestock, such as breeding and slaughtering.

The results of our temporal cluster analysis show that there is a distinct seasonality in human brucellosis. The main cluster time of the reported cases is from March to August, the peak incidence occurred in early spring to early summer, and the lowest incidence usually occurred in winter. This result is consistent with the results from other studies in China [41–43] and some other Asian countries [44, 45]. The reason might be that these months represent the time of lambing in the agricultural and pastoral areas. Since Brucella exists in tissues and body fluids in the placenta, fetal membranes,

amniotic fluid, breasts, and lymph nodes. during the breeding seasons, the risk of exposure to Brucella increases correspondingly [41]. It may also be due to warm temperatures suitable for the transmission of zoonosis, according to some reported research findings, the factors of temperature, sunshine, wind, speed, altitude and rainfall will affect the introduction of brucellosis [46, 47]. Identifying the peak of seasonal incidence can be used to strengthen prevention and control strategies before the incidence increases.

The results of spatial autocorrelation analysis showed that the incidence of brucellosis in Shandong Province from 2015 to 2021 had a certain spatial clustered with positive spatial autocorrelation at the county level. The Moran’s I value of the global spatial autocorrelation index decreased first and then increased, indicating that the degree of global spatial clustered decreased first and then increased, the highest clustering degree was in 2020.Local autocorrelation analysis results were showed that the “high-high” clustered area was mainly concentrated in the northern part of the province. Located in distributed in Hekou District, Lijin County, Kenli District, Wudi County, Yangxin County, Huimin County, Zhanhua County, Bincheng District, Qingyun County, Shanghe County, Pingyi County, Shouguang city, Sishui County, Qufu City, Xintai City, Ningyang County and other regions. Hekou District, Lijin County and Zhanhua County are the “high-high” gathering areas over the years. It may be due to the large number of animal husbandry and animal product processing industries in these areas, there are many workers in these related industries, the backward traditional breeding methods, the poor behavior of family captivity, the mixed living of human and animal, and the weak awareness of protection, leading to more opportunities for infection. Over time, the number of counties (districts) covered by high-high clustered areas showed a downward trend. In recent years, Pingyi County of Linyi City, Ningyang County of Tai ‘an city and Qufu City of Jining became the new “high-high” clustered area. The range of “low-low” clustered had been shrinking year by year, and the new “high-high” clustered appears in the eastern region. Be alert to these new “high-high” clusters and strengthen targeted prevention and control measures.

The Spatio temporal cluster analysis identified one most likely cluster, one secondary cluster and one 2nd secondary cluster of human brucellosis incidence. The most likely cluster was located in the northern areas, including 37 counties and districts, which indicated that the prevention and control measures in these regions still need to be strengthened. The secondary clusters were distributed in the southern area, including Qufu City, Sishui County, mainly ranged from January 2019 to December 2021.This means that brucellosis moves from north to south; thus, prevention and control awareness should also be established in these areas. This Spatio-temporal cluster study of brucellosis helps to identify high-risk areas and time frames for brucellosis, to provide basis for government decision-making. According to the results of our study, we suggest to further strengthen surveillance and prevention and control measures in the southern region.

Direct contact with the infectious livestock remains the primary source of human brucellosis. the increasing demand for mutton and beef with the improvement of living standards, which has led to an increase in the transportation of cattle, sheep, and other animal products from the northern pastoral areas [48]. Inter-provincial movement of livestock products increase exposure to infected animals and contaminated livestock products. It is critical to strengthen the management and supervision of the agricultural market and the interprovincial transportation of livestock to prevent contaminated meat and dairy products from entering the market. It is suggested that the animal husbandry supervision department should strengthen the quarantine of cattle and sheep and the supervision of livestock circulation and transportation to prevent the flow of diseased animals and their livestock products.

Governments at all levels should establish joint prevention and control mechanism involving multiple departments among high-risk areas. Relevant departments in different regions should continue to strengthen both the prevention and control throughout the year, to contain the spread of brucellosis. Brucellosis is a reemerging disease for both humans and animals [49], health and animal husbandry departments should strengthen cooperation, strengthen information exchange and communication, regularly inform each other of the epidemic situation, and ensure that all prevention and control measures are in place. Increase investment in brucellosis prevention and control in key areas, strengthen training of medical personnel, effectively improve the diagnosis and treatment level of brucellosis in medical institutions, earnestly do a good job in the diagnosis and reporting of brucellosis cases, achieve early detection, diagnosis, reporting and treatment, standardize treatment, and reduce the chronic brucellosis.

Strengthen information dissemination and health education of brucellosis among key groups, such as family breeder, intervention packages have been issued to guide livestock breeding and livestock product production and processing occupational groups to do a good job in personal protection. Health education videos and leaflets has been used to increase the intensity and frequency of education and improve the protection awareness of employees. Comprehensive prevention and control measures to reduce brucellosis infection rate, to ensure the health of the people.

## Conclusion

Brucellosis continues to be a common challenge in Shandong province, especially for counties in the northern region from March to August. In such higher-risk susceptible areas, more disease prevention and control measures should be taken to reduce the incidence of brucellosis and ensure the health of the people. Further researches should focus more on the influencing factors, such as the environmental and socio-economic factors, to provide more guidelines for policy makers to initiate control strategies.

## Data Availability

The datasets generated during and analyzed during the current study are not publicly available but are available from the corresponding author on reasonable request.

## Abbreviations

LLR: Likelihood Rate
RR: Relative Risk
LISA: Local indicators of spatial association
